# Adiponectin promotes pancreatic cancer progression by inhibiting apoptosis via the activation of *AMPK/Sirt1/PGC-1α* signaling

**DOI:** 10.18632/oncotarget.1963

**Published:** 2014-05-13

**Authors:** Bingqing Huang, Xixi Cheng, Dan Wang, Meiyu Peng, Zhenyi Xue, Yurong Da, Ning Zhang, Zhi Yao, Min Li, Aimin Xu, Rongxin Zhang

**Affiliations:** ^1^ Research Center of Basic Medical Science, Tianjin Medical University, Tianjin, China; ^2^ Key Laboratory of Immune Microenvironment and Diseases of Educational Ministry of China, Department of Immunology, Basic Medical College, Tianjin Medical University, Tianjin, China; ^3^ Department of Integrative Biology & Pharmacology, The University of Texas Medical School at Houston, Houston, TX, USA; ^4^ State Key laboratory of Pharmaceutical Biotechnology, and Department of Medicine, University of Hong Kong, Hong Kong, China

**Keywords:** Adiponectin, AMPK, Sirt1, PGC1α, Apoptosis, Pancreatic cancer

## Abstract

Adiponectin is an adipocyte-secreted adipokine with pleiotropic actions. Clinical evidence has shown that serum adiponectin levels are increased and that adiponectin can protect pancreatic beta cells against apoptosis, which suggests that adiponectin may play an anti-apoptotic role in pancreatic cancer (PC). Here, we investigated the effects of adiponectin on PC development and elucidated the underlying molecular mechanisms. Adiponectin deficiency markedly attenuated pancreatic tumorigenesis in vivo. We found that adiponectin significantly inhibited the apoptosis of both human and mouse pancreatic cancer cells via adipoR1, but not adipoR2. Furthermore, adiponectin can increase AMP-activated protein kinase (AMPK) phosphorylation and NAD-dependent deacetylase sirtuin-1 (Sirt1) of PC cells. Knockdown of AMPK or Sirt1 can increase the apoptosis in PC cells. AMPK up-regulated Sirt1, and Sirt1 can inversely phosphorylate AMPK. Further studies have shown that Sirt1 can deacetylate peroxisome proliferator-activated receptor gamma coactivator 1-alpha (PGC1α), which can increase the expression levels of mitochondrial genes. Thus, adiponectin exerts potent anti-apoptotic effects on PC cells via the activation of AMPK/Sirt1/PGC1α signaling. Finally, adiponectin can elevate β-catenin levels. Taken together, these novel findings reveal an unconventional role of adiponectin in promoting pancreatic cancers, and suggest that the effects of adiponectin on tumorigenesis are highly tissue-dependent.

## INTRODUCTION

Pancreatic cancer, which is a gastrointestinal malignancy with increasing incidence and mortality rates, exhibits dismal prognosis and no effective treatment, with a 5-year survival rate of less than 5%. Approximately 90% of pancreatic cancers originate in the duct of the glandular epithelium, which indicate that pancreatic cancer has a strong ability to metastasize [1, 2]. Many studies have shown that there is a correlation between obesity and cancer, although the precise mechanisms underlying this relationship remain obscure [3-5]. Adiponectin, which is encoded by the Adipoq gene, is an anti-diabetic and anti-inflammatory adipokine, and its plasma concentration is decreased in obesity [3]. Adiponectin has two receptors, AdipoR1 and AdipoR2, both of which are highly expressed in tumor tissues of pancreatic cancer [2].

Many case-control and prospective studies have shown that the serum concentration of adiponectin is decreased in breast cancer, hepatocellular carcinoma (HCC) and colorectal cancer [6-8]. In breast cancer, adiponectin can inhibit cell proliferation and invasion by decreasing phospho-AKT/GSK3β and β-catenin levels or activating the AMPK–S6K axis. Adiponectin inhibits hepatocellular carcinoma by increasing phosphorylation of c-Jun-N-terminal kinase (JNK) and inhibiting mammalian target of rapamycin (mTOR) phosphorylation [9]. Adiponectin also induces anti-angiogenesis and antitumor activity in sarcoma [10]. However, the association of serum adiponectin and pancreatic cancer remains controversial. One prospective study reported that the median plasma adiponectin concentration is lower in case subjects compared with control subjects [11]. In contrast, many other studies found higher serum adiponectin levels or a higher adiponectin/leptin ratio in pancreatic cancer patients who exhibited a positive or strong positive expression of AdipoR1 and AdipoR2 and that this higher level correlated strongly with the proinflammatory cytokine IL-6 [2, 12-14]. Importantly, adiponectin can protect against pancreatic beta cell apoptosis by the induction of Erk and Akt phosphorylation [15]. Taken together, these foundings suggested that adiponectin may play an anti-apoptotic role in pancreatic cancer, and thus requires further exploration.

In mechanistic studies, there is some indirect evidence that also shows that adiponectin may unconventionally regulate several proliferative signaling pathways under specific conditions [16, 17]. Barb et al. found that adiponectin can activate the mTOR pathway in PTEN-deficient cells and that this adiponectin stimulation of mTOR is mediated via AKT activation [16]. Habeeb et al. found that adiponectin inhibits colon cancer cell growth in glucose-containing medium, but can support cell survival in glucose-deprived medium via the induction of autophagy [17]. These findings suggested that adiponectin plays distinct roles in different environments and cancers. It has been reported that adiponectin can increase mitochondrial biogenesis and attenuate mitochondrial disorders via the activation of AMPK/PGC1α in the diabetic heart [18]. In melanoma cells, activation of PGC1α can increase mitochondrial energy metabolism and reactive oxygen species (ROS) detoxification capacities and reprogram cell metabolism to maintain cell growth and survival [19]. The metabolism and secretory activities of the pancreas are highly exuberant. Compared with other cancers, pancreatic cancer utilizes a noncanonical pathway of glutamine to increase the NADPH/NADP^+^ ratio, which can potentially maintain the cellular redox state [20]. Given the anti-apoptotic effect of adiponectin on the pancreas and heart [21], we hypothesized that adiponectin may increase mitochondrial biogenesis and inhibit apoptosis of pancreatic cancer cells via AMPK/Sirt1/PGC1α. Consistent with our expectations, our in vitro and in vivo studies revealed that adiponectin can strongly promote pancreatic cancer cell growth via the inhibition of apoptosis, and we simultaneously investigated the underlying molecular mechanisms.

## RESULTS

### Adiponectin deficiency inhibits pancreatic cancer cell growth and metastasis in C57BL/6 mice

**Figure 1:**
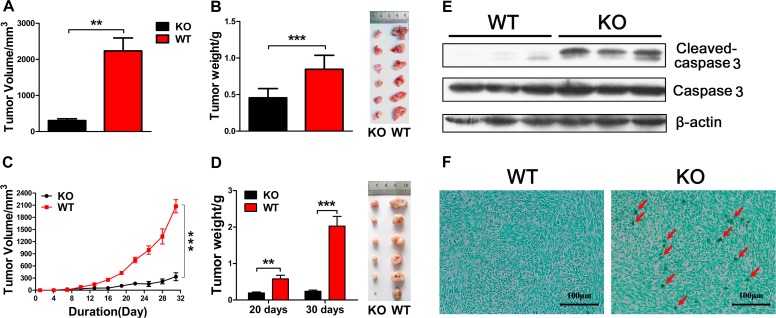
Adiponectin deficiency inhibits pancreatic cancer cell growth in vivo (A, B) 5×10^5^ H7 cells were injected into the orthotopic pancreas per mouse (WT: n=8, KO: n=6). The mice were sacrificed two weeks after inoculation and the tumors were collected, measured, weighed and imaged. (C, D) WT and APN KO mice were subcutaneously challenged with H7 cells (1×10^6^ cells per mice, n=16 per group) in the flank. The tumor size of H7 cells was monitored every three days for 30 days. Twenty and 30 days after the inoculation of H7 cells, the mice were sacrificed, and the tumors were collected, weighed and imaged. (E) H7 tumor lysates from APN KO and WT mice were analyzed using immunoblotting with the indicated antibodies. (F) Representative TUNEL stained H7 tumor section from APN KO and WT mice. The values represent the mean ± SEM of three independent experiments performed in triplicate. **: 0.001<p<0.01; ***: p<0.001.

H7 and Panc02 mouse pancreatic cancer cell lines were derived from a C57BL/6 background [22, 23]. To examine the role of adiponectin in the regulation of pancreatic cancer growth, orthotopic inoculation of H7 cells was performed in adiponectin (APN) -KO and WT mice. The size and weight of the resulting tumor were measured. Tumors from WT mice were notably larger and heavier compared to the tumors from APN KO mice (Figure 1A, B). Unexpectedly, adiponectin deficiency significantly reduced the metastasis of H7 cells in the intestine, lung, and kidney (Supplementary Figure S1A, B, and C). H&E staining of tissue sections more clearly confirmed these results (Supplementary Figure S1D). On the basis of these results, we concluded that adiponectin plays an unorthodox role in the regulation of pancreatic cancer growth and metastasis.

To further confirm the relationship between adiponectin and pancreatic cancer, we subcutaneously injected H7 and Panc02 cells into APN KO and WT mice. H7 and Panc02 cells were able to grow in both APN KO and WT mice, and there was a significant reduction in tumor size and tumor weight in APN KO mice, which suggested that adiponectin could promote pancreatic cancer growth *in vivo* (Figure 1C, D, Supplementary Figure S2A, B). We used quantitative PCR and ELISA to further determine the authenticity of the APN knockout mice. There is nearly no expression of adiponectin in APN KO mice compared with WT mice (Supplementary Figure S2C, D). Taken together, these results suggested that adiponectin promoted pancreatic cancer cell growth.

### Adiponectin exhibits an anti-apoptotic role in pancreatic cancer

**Figure 2:**
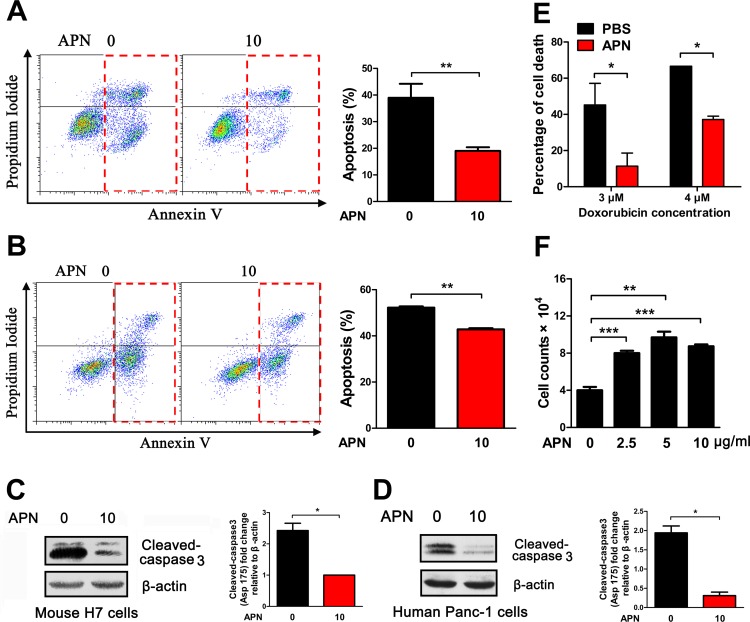
Adiponectin exerts anti-apoptotic effects in mouse and human pancreatic cancer cells (A) H7 cells were treated with adiponectin (0 or 10 μg/ml) and doxorubicin (2 μM) in 2% FBS medium for 24 hours, and apoptosis was then detected using Annexin V. (B) Apoptosis of Panc-1 cells after treatment with adiponectin (0 or 10 μg/ml) and doxorubicin (6 μM) in 2% FBS medium for 24 hours. (C, D) The cells were treated as described in A and B, and the levels of cleaved- caspase 3 in H7 and Panc-1 cells were analyzed using immunoblotting. (E) Cell death assay results. Twenty-four hours after APN and doxorubicin treatment, the number of dead cells of H7 was quantified and expressed as the percentage of total cells. (F) Cell proliferation assay results. Twenty thousand H7 cells were plated in 12-well plates and treated with adiponectin (1-10 μg/ml) and doxorubicin (2 μM) in 2% FBS medium for 48 hours, and then quantification. The cell number from three independent experimental wells was plotted. The values represent the mean ± SEM of three independent experiments performed in triplicate. *: 0.01<p<0.05; **: 0.001<p<0.01.

It has been proposed that adiponectin can reduce apoptosis in the heart and pancreas via noncanonical pathways [15, 21, 24]. Given the role of adiponectin in the promotion of pancreatic cancer cell growth, we investigated whether adiponectin promoted pancreatic cancer growth by inhibiting apoptosis of pancreatic cancer cells. Adiponectin can significantly inhibit the apoptosis of pancreatic cancer *in vivo* by decreasing the levels of the apoptotic marker cleaved-caspase 3 (Figure 1E). TUNEL-stained H7 tumor sections were consistent with Western blotting results (Figure 1F). To determine whether adiponectin can protect pancreatic cancer cells against apoptosis in vitro, mouse H7 and human Panc-1 pancreatic cancer cell lines were directly treated with adiponectin and doxorubicin. We found that adiponectin inhibited the apoptosis induced by doxorubicin in both H7 and Panc-1 cells (Figure 2A, B). This decreased apoptosis was mediated via inhibition of cleaved-caspase 3 expression (Figure 2C, D). To further confirm the biological activity of adiponectin, we treated HepG2 (human HCC cell lines) cells with adiponectin and found that adiponectin increased apoptosis induction in HepG2 cells by more than one-fold (Supplementary Figure S3A, B). Western blotting analyses showed that adiponectin could increase the levels of cleaved-caspase 3 in HepG2 cells (Supplementary Figure S3C, D) [9]. Moreover, adiponectin can decrease pancreatic cancer cell death and increase proliferation (Figure 2E, F). Taken together, these results suggested that adiponectin could decrease the apoptosis and death of pancreatic cancer cells via the suppression of caspase 3 activation.

### Adiponectin-induced anti-apoptosis effects are mediated by AdipoR1 and not AdipoR2

**Figure 3:**
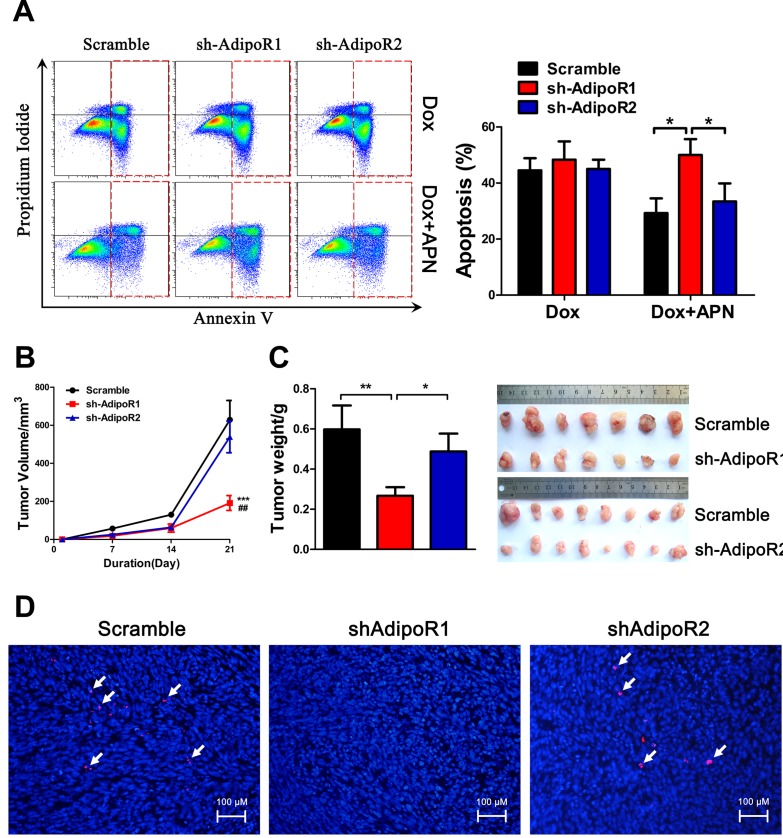
Suppression of AdipoR1 promotes apoptosis and inhibits proliferation (A) Annexin V analysis of apoptosis after AdiopR1/2 knockdown in H7 cells treated with adiponectin (10 μg/ml) or doxorubicin (2 μM) in 2% FBS medium for 24 hours. (B, C) Tumor formation assay results after knockdown of AdipoRs. 1×10^6^ H7 cells with the indicated expression conditions of AdipoRs were injected subcutaneously into 6- to 8- week-old C57BL/6 mice. The tumor size was monitored once a week for three weeks. Next, the mice were sacrificed, and the tumors were resected, measured and weighed (n=8 mice per group, ***: shAdipoR1 and Scramble; ##: shAdipoR1 and shAdipoR2). (D) Immunofluorescence of Ki-67 in H7 tumor sections after AdipoR knockdown. Scale bars, 100 μm. The values represent the mean ± SEM of three independent experiments performed in triplicate. *: 0.01<p<0.05; ** or ##: 0.001<p<0.01; ***: p<0.001.

To determine the role of AdipoR in pancreatic cancer growth, we first tested the expression of AdipoRs using RT-PCR in H7 and Panc02 cell lines and found that both cell lines expressed AdipoR1 and AdipoR2. AdipoR1 expression levels were relatively higher compared to AdipoR2 in both cell lines (Supplementary Figure S4A, B). Next, we suppressed AdipoR1 or AdipoR2 expression (Supplementary Figure S4C-F) and found that knockdown of AdipoR1, but not that of AdipoR2, resulted in a 2.5-fold increase in the percentage of apoptotic H7 cells (Figure 3A).

To further investigate the role of AdipoRs, AdipoR1 or AdipoR2 knockdown H7 or Panc02 cells and scramble cells were subcutaneously injected into C57BL/6 mice. Knockdown of AdipoR1 in H7 and Panc02 cells significantly reduced tumor size and tumor weight, suggesting that AdipoR1 was more important for adiponectin promotion of pancreatic cancer growth (Figure 3B, C, Supplementary Figure S4G, H). We also detected the effect of shRNA on the targeting AdipoRs following *in vivo* studies, and found that the expression levels of both AdipoRs were effectively inhibited (Supplementary Figure S5A-D). In addition, AdipoR1 knockdown markedly decreased the expression of Ki-67, which indicated that AdipoR1 was indispensable for adiponectin-induced proliferation of pancreatic cancer cells (Figure 3D, Supplementary Figure S4I). Taken together, these results demonstrated that suppression of AdipoR1, but not AdipoR2, activates the intrinsic apoptotic pathway.

### AMPK is a pivotal mediator of adiponectin-suppression of caspase 3 activity

**Figure 4:**
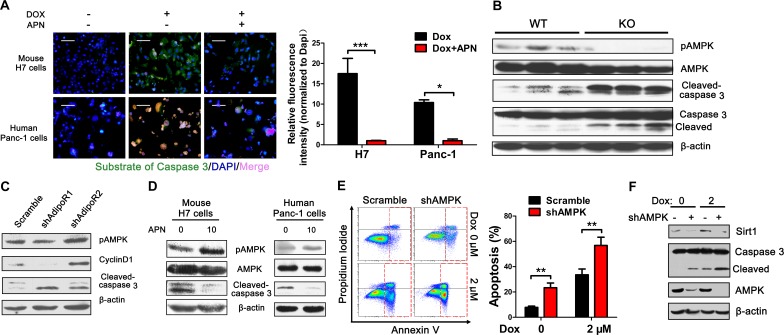
Adiponectin-induced AMPK phosphorylation is essential for the anti-apoptotic effect (A) Fluorescence demonstrating the caspase 3 activity of mouse H7 or human Panc-1 cells treated with full-length mouse or human adiponectin (0 or 10 μg/ml) combined with doxorubicin (2 μM or 6 μM) for 24 hours. Scale bars, 50 μm. (B) H7 tumor lysates from APN KO and WT mice were analyzed using immunoblotting with the indicated antibodies. (C) Western blotting analysis of pAMPK, cleaved-caspase 3, cyclinD1 in H7 cells stably expressing control or AdipoR1/2 shRNA. (D) Mouse H7 or human Panc-1 cells were treated with full-length mouse or human adiponectin (0 or 10 μg/ml) combined with doxorubicin (2 μM or 6 μM) for 24 hours. Total protein lysates were analyzed using immunoblotting with the indicated antibodies. (E) Annexin V analysis of apoptosis after AMPK knockdown in H7 cells treated with doxorubicin (0 or 2 μM). (F) Western blotting analysis of Sirt1 and Caspase 3 in H7 cells stably expressing control or AMPK shRNA. The values represent the mean ± SEM of three independent experiments performed in triplicate. *: 0.01<p<0.05; **: 0.001<p<0.01.

To further investigate the anti-apoptotic effect of adiponectin on pancreatic cancer cells, we next evaluated the activity of caspase 3 of pancreatic cancer cells after treatment with adiponectin using a new experimental technology [25]. Adiponectin significantly suppressed caspase 3 activity induced by doxorubicin in pancreatic cancer cells (Figure 4A). AMPK has been hypothesized to be a crucial sensor of energy status and plays important pleiotropic roles in cellular responses to metabolic stress [26-28]. To investigate the role of AMPK in pancreatic cancer growth regulated by adiponectin, H7 tumor lysates obtained from APN KO and WT mice were detected using Western blotting analyses with anti-phospho-AMPK antibody. Adiponectin significantly increased AMPK phosphorylation, and this effect was accompanied with decreased levels of cleaved-caspase 3, suggesting that adiponectin inhibited the apoptosis of pancreatic cancer cells most likely via AMPK (Figure 4B). These results were consistently found in our *in vitro* studies. Furthermore, AdipoR1 knockdown resulted in a more than a 2-fold inhibition of AMPK phosphorylation, significant activation of caspase 3, and down-regulation of cyclinD1 (Figure 4C). To investigate the role of AMPK in pancreatic cancer regulated by adiponectin, H7 and Panc-1 cells lysates treated with adiponectin were detected using immunoblotting analyses and showed higher levels of phospho-AMPK and lower levels of cleaved-caspase 3 (Figure 4D). AMPK knockdown resulted in a more than 3-fold induction in the percentage of apoptotic cells in H7 cells (Figure 4E), and this result was consistent with western blotting results (Figure 4F). Taken together, these results suggested that AMPK plays an important role in regulating pancreatic cancer cell apoptosis via adiponectin.

### Adiponectin inhibits apoptosis of pancreatic cancer cell via activation of AMPK-Sirt1-PGC1α signaling

**Figure 5:**
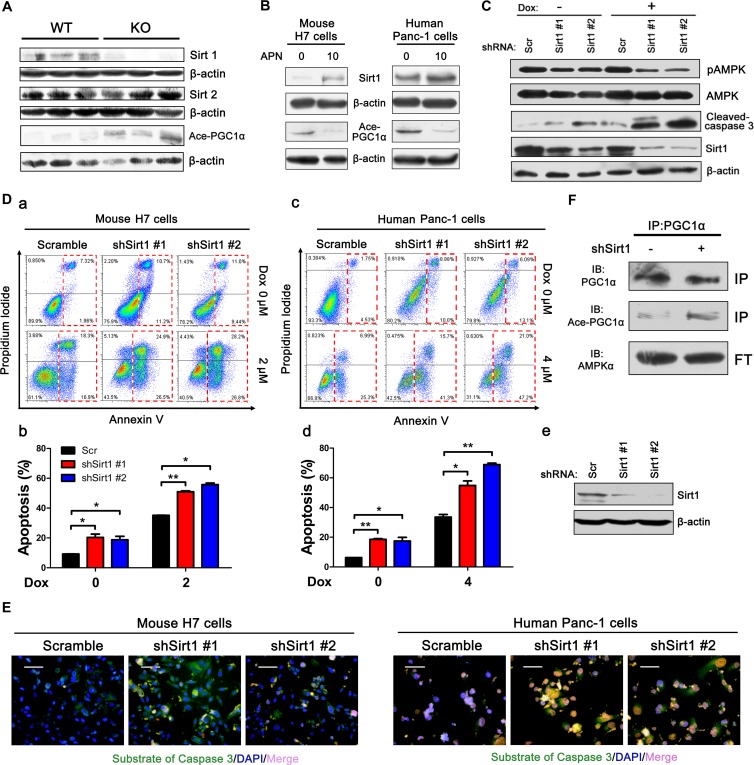
Adiponectin modulates the AMPK-Sirt1-PGC1α axis (A) H7 tumor lysates from APN KO and WT mice were analyzed using immunoblotting with the indicated antibodies. (B) Mouse H7 or human Panc-1 cells were treated with full-length mouse or human adiponectin (0 or 10 μg/ml) combined with doxorubicin (2 μM or 6 μM) for 24 hours. Total protein lysates were analyzed using immunoblotting with the indicated antibodies. (C) Western blotting analysis of pAMPK, AMPK, cleaved-caspase 3 in H7 cells stably expressing control or Sirt1 shRNA. (D) Annexin V analysis of apoptosis after Sirt1 knockdown in H7 cells treated with doxorubicin (2 μM) or Panc-1 cells treated with doxorubicin (6 μM) for 12 hours. (a, c) Annexin V diagram. (c, d) quantification of the percentage of apoptotic cells using the Annexin V assay. (e) Western blotting analysis of Sirt1 levels in Panc-1 cells. (E) Fluorescence demonstrated the caspase 3 activity of mouse H7 cells treated with doxorubicin (2 μM) or human Panc-1 cells treated with doxorubicin (6 μM) for 12 hours after Sirt1 knockdown. Scale bars, 50 μm. (F) Sirt1 knockdown H7 cells were harvested and subjected to immunoprecipitation using anti-PGC1α antibody, and western blots were performed for the indicated proteins. The values represent the mean ± SEM of three independent experiments performed in triplicate. *: 0.01<p<0.05; **: 0.001<p<0.01.

Consistent with previous studies [26, 29, 30], we found that AMPK knockdown down-regulated Sirt1 (Figure 4F). Sirt1, which belongs to the family of sirtuins, is a protein deacetylase that is dependent on nicotine adenine dinucleotide (NAD) [31, 32]. Several studies have shown that inhibition of Sirt1 and Sirt2 in human pancreatic cancer cell lines can significantly inhibit cell proliferation and induce apoptosis [33-35]. Clinical epidemiological studies have also found that Sirt1 is overexpressed in pancreatic cancer tissues at both the mRNA and protein levels compared with adjacent normal pancreatic tissues [34, 36]. Adiponectin deficiency can significantly decrease the level of Sirt1 but has no effect on Sirt2, which suggests that inhibition of apoptosis by adiponectin is dependent on Sirt1 and not on Sirt2 (Figure 5A). The *in vitro* results further showed that adiponectin can up-regulate Sirt1 levels in both H7 and Panc-1 cells (Figure 5B). Sirt1 knockdown resulted in higher apoptosis in H7 and Panc-1 cells (Figure 5C, D) and Sirt1 knockdown also activated the caspase 3 activity (Figure 5E). We found that AMPK can modulate Sirt1 levels (Figure 4F). Expectedly, Sirt1 knockdown also decreased AMPK phosphorylation (Figure 5C).

Peroxisome proliferator-activated receptor gamma coactivator 1-alpha (PGC1α) is a regulator of mitochondrial biogenesis and function [19, 37]. It has been reported that AMPK-Sirt1-PGC1α is an important energy-sensing metabolic pathway [38]. We found that tumor cells from APN KO mice exhibited increased acetylation levels of PGC1α, which suggested that adiponectin deficiency can inhibit the activity of PGC1α (Figure 5A, B). To test if Sirt1 can deacetylate PGC1α, immunoprecipitation was performed using anti-PGC1α antibody. Consistent with expectations, Sirt1 knockdown increased the acetylation levels of PGC1α (Figure 5F). Taken together, these results suggested that adiponectin could activate AMPK-Sirt1-PGC1α signaling.

### Adiponectin enhances mitochondrial gene expression via PGC1α

**Figure 6:**
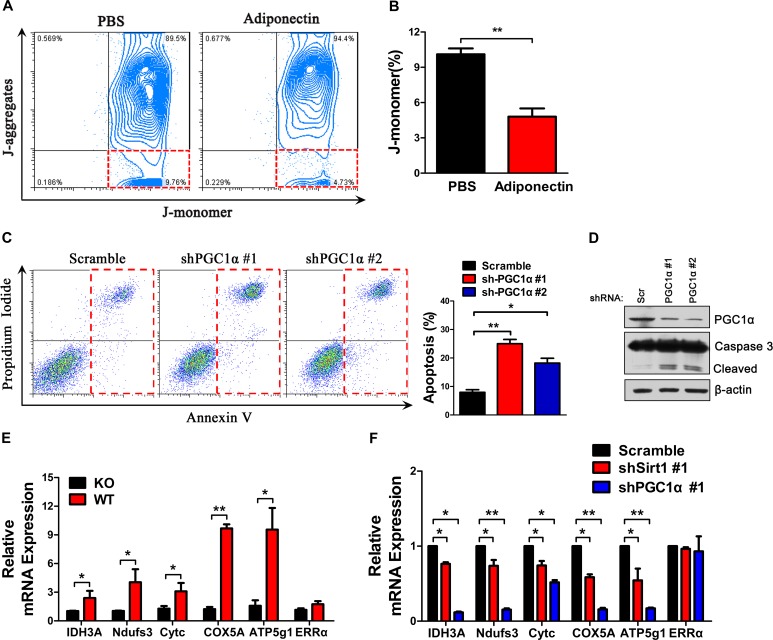
Adiponectin protects the functional integrity of the mitochondrial membrane, and exerts potent anti-apoptotic effects (A) Mitochondrial membrane potential detection. H7 cells were treated with full-length mouse adiponectin (0 or 10 μg/ml) combined with doxorubicin (2 μM) for 24 hours and measured using the JC-1 dye. Healthy mitochondria were polarized, and JC-1 was rapidly taken up by these mitochondria to form JC-1 aggregates. When the mitochondrial membrane was damaged, JC-1 does not accumulate in the mitochondria and flows into the cytoplasm as monomers. (B) Quantification of the percentage of J-monomer. (C) Annexin V analysis of apoptosis after PGC1α knockdown in H7 cells. (D) Western blotting analysis of cleaved-caspase 3 in PGC1α knock-down and control H7 cells. (E) mRNA expression levels of mitochondrial genes in H7 tumor cells from APN KO and WT mice. (F) mRNA expression levels of mitochondrial genes in Sirt1 or PGC1α knockdown H7 cells. The values represent the mean ± SEM of three independent experiments performed in triplicate. *: 0.01<p<0.05; **: 0.001<p<0.01.

Consistent with its anti-apoptosis effect, adiponectin-treated H7 cells showed a lower decrease in mitochondrial membrane potential, as determined using the J-aggregation fluorescence assay (Figure 6A, B). We further investigated the anti-apoptotic role of PGC1α activated by adiponectin in pancreatic cancer cells and found that PGC1α knockdown resulted in a 2.5-fold increase in the percentage of apoptotic H7 cells, which was consistent with Western blotting results (Figure 6C, D). Expectedly, adiponectin deficiency resulted in a decrease in the levels of mitochondrial genes (Figure 6E). Sirt1 or PGC1α knockdown also resulted in a decrease in the levels of mitochondrial genes (Figure 6F). Taken together, these results suggested that adiponectin can protect the mitochondrial membrane potential and increase the expression of mitochondrial genes via the activation of PGC1α.

### Inhibition of caspase 3 modulates the degradation of β-catenin and increases the expression of cyclinD1

**Figure 7:**
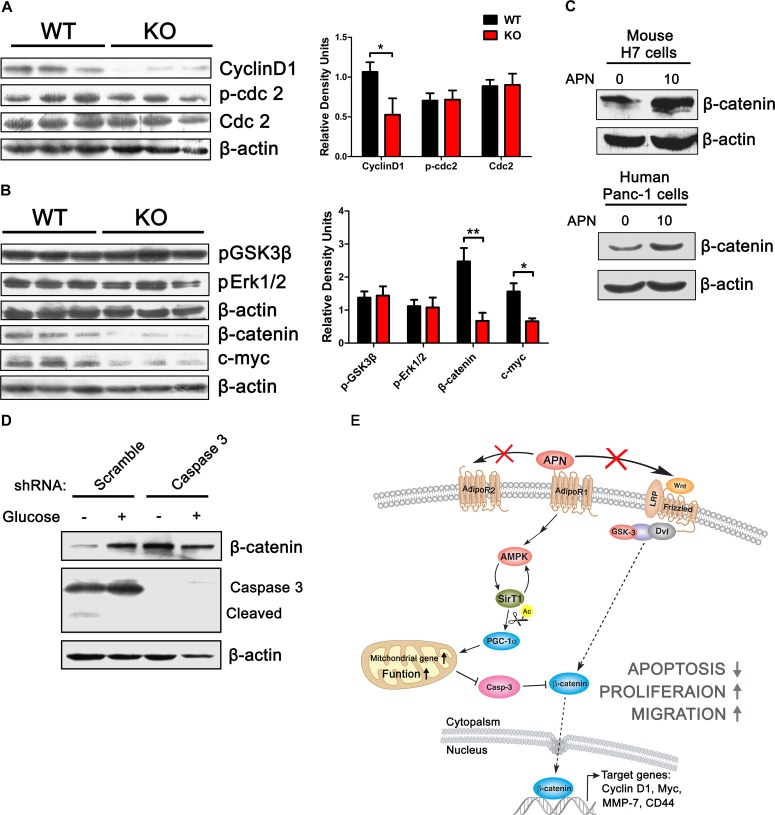
Adiponectin can elevate β-catenin and cyclinD1 levels (A, B) H7 tumor lysates from APN KO and WT mice were analyzed using immunoblotting with the indicated antibodies. (C) Western blotting analysis of H7 cells treated as shown in Figure 2A using the indicated antibodies. (D) Western blotting analysis of β-catenin, caspase 3 in H7 cells stably expressing control or caspase 3 shRNA after treatment with glucose (0 or 25 mM) for 24 hours. The values represent the mean ± SEM of three independent experiments performed in triplicate. *: 0.01<p<0.05; **: 0.001<p<0.01. (E) A model based on our studies. In pancreatic cancer, adiponectin protects the functional integrity of the mitochondrial membrane via the activation of AMPK-Sirt1-PGC1α signaling and exerts potent anti-apoptotic effects. Decreased apoptosis inhibits the degradation of β-catenin, and promotes the cyclinD1 expression.

We found that adiponectin can promote H7 cell proliferation in a dose-dependent manner (Figure 2F). Next, we investigated the mechanism responsible for this increase in cell number after treatment with adiponectin and found that tumor cells from APN KO mice expressed less cyclinD1 compared to WT mice (Figure 7A). CyclinD1 is responsible for the cell cycle progression from the G1 to S phase, where the cell cycle transition from the G2 to the M phase is controlled by cyclinB [39, 40]. Cdc-2 and its phosphorylation status, which are coordinated with cyclinB, exhibited no change between APN KO and WT mice (Figure 7A), which indicated that adiponectin promoted pancreatic cancer growth via the induction of cell cycle transition from the G1 to S phase. It is known that β-catenin can bind to the cyclinD promoter and activate its expression [41]. The total level of β-catenin and c-myc in H7 tumors from APN KO mice is lower compared to that obtained from WT mice, which was consistent with the results of the *in vitro* study (Figure 7B, C).

β-catenin is a central effector in Wnt signaling. [42]. However, we found that adiponectin has not effect on the phosphorylation of GSK3β, Wnt3a and Wnt5a (Figure 7B, Supplementary Figure S6A, B). Adiponectin also has no effect on the activity of Erk (Figure 7B, Supplementary Figure S6B), which suggests that there is a noncanonical regulatory pathway for β-catenin in pancreatic cancer cells. Numerous studies have shown that caspase 3 can induce the degradation of β-catenin independent of GSK3β and Erk activity [43, 44]. We found that a reverse regulatory relationship exists between caspase 3 and β-catenin in H7 cells (Figure 7D). In addition, glucose starvation-induced caspase 3 activation could decrease the level of β-catenin, and deletion of caspase 3 can decrease the degradation of β-catenin (Figure 7D). Taken together, these results suggested that upregulation of β-catenin and cyclinD1 by adiponectin is mediated via a decrease in the apoptosis of pancreatic cancer cells.

## DISCUSSION

Although it has been reported that adiponectin plays a positive role in many cancers, the relationship between adiponectin and pancreatic cancer remains unclear [9, 10, 42, 45]. Here we provide the first evidence that adiponectin KO mice exhibit growth inhibition of pancreatic cancer cells.

Unlike most other cancers, our *in vivo* studies showed that adiponectin deficiency could significantly alleviate pancreatic cancer growth and metastasis, suggesting that there is an unconventional role of adiponectin in the promotion of pancreatic cancer progression. Further experiments showed that adiponectin inhibited the apoptosis of pancreatic cancer cells via AdipoR1, which was consistent with the effect on myocytes. In hepatocellular carcinoma, adiponectin induced apoptosis via activation of the AMPK-JNK-caspase3 pathway [9]. Adiponectin stimulated AMPK and extracellular Ca^2+^ influx, which were involved in glucose and lipid metabolism partially via mitochondrial biogenesis in cardiac myocytes, pancreatic beta cells, and the liver [24]. To investigate whether the anti-apoptotic effect of adiponectin in pancreatic cancer cells is mediated by AMPK, we detected the phosphorylation level of AMPK in AdipoR-knockdown H7 cells treated with adiponectin and found that AdipoR1 knockdown resulted in a decreased level of AMPK phosphorylation accompanied with an increase in caspase 3 activation. AMPK plays a distinct role in pancreatic cancer cells, which is consistent with our expectations. We suspected that pancreatic cancer cells might reprogram their metabolism via AMPK signaling to meet the anabolic and energetic demands necessary for growth and survival.

AMPK can upregulate Sirt1 and induce deacetylation of PGC1α [18, 38]. It has been reported that PGC1α can reprogram cancer cell metabolism and enhance mitochondrial biogenesis via the activation of a series of mitochondrial genes [19]. Our studies showed that adiponectin can upregulate Sirt1 levels, and Sirt1 can conversely phosphorylate AMPK. The levels of mitochondrial genes were increased to different levels in H7 tumors cells from WT mice compared with KO mice, which was comfirmed by Sirt1 or PGC1α knockdown. Moreover, PGC1α knockdown in H7 cells induced higher levels of apoptosis compared with control cells and resulted in a significant activation of caspase 3. These results revealed that adiponectin protected pancreatic cancer cells against apoptosis via the activation of PGC1α and mitochondrial genes. Holland et al. reported that adiponectin can significantly reduce hepatic ceramide levels and blood glucose levels via the activation of ceramidase activity, which can phosphorylate AMPK through the activation of calcium influx and protect pancreatic beta cells against apoptosis [46]. However, it is still unclear whether adiponectin can activate the AMPK-Sirt1-PGC1α signaling axis via the activation of ceramidase activity in pancreatic cancer.

Our results have also shown that adiponectin was able to induce the cell cycle transition from the G1 to S phase via the upregulation of cyclinD1 expression, which was transcriptionally activated by β-catenin, but did not affect the transition from the G2 to M phase in H7 cells. Several studies have reported that adiponectin can decrease the phosphorylation of GSK3β and increase the phosphorylation of β-catenin, which resulted in its degradation [42, 47, 48]. Other research studies have found that adiponectin-induced Erk and AKT phosphorylation protected against pancreatic beta cell apoptosis and ameliorated doxorubicin-induced cardiotoxicity [15, 49]. Erk has been shown to increase the transcriptional activity of β-catenin via a Wnt-dependent or -independent pathway [50, 51]. Unexpectedly, the phosphorylation levels of AKT, Erk1/2 and GSK-3β were not all affected by adiponectin in pancreatic cancer cells, which suggested that an unconventional pathway regulates β-catenin. We found that decreased apoptosis can upregulate β-catenin, which can be blocked by the deletion of caspase 3 in H7 cells [43, 44]. We proposed that these apoptotic cells might activate the ubiquitin-conjugating enzyme and target β-catenin for degradation via the proteasome. In addition, we found that adiponectin deficiency significantly inhibited the metastasis of H7 cells, which is a highly metastatic cell line derived from the Panc02 cell line. The mechanism underlying this inhibition of metastasis by adiponectin remains to be explored.

In summary, this study revealed that adiponectin plays a role in inhibition of an intrinsic apoptotic pathway and promotion of cell survival in pancreatic cancer. Adiponectin exerted potent anti-apoptotic effects via the activation of AMPK/Sirt1/PGC1α signaling. Taken together, these findings suggested that the effects of adiponectin on tumorigenesis are highly tissue-dependent and different cancers should use different treatment strategies to target adiponectin.

## MATERIALS AND METHODS

### Mice and animal studies

Female 6- to 8-week-old C57BL/6 mice were purchased from the Academy of Military Medical Science (Beijing, China). Adiponectin knock-out mice (Adipoq^−/−^) in a C57BL/6 background were generated at the laboratory of Lawrence Chan (Baylor College, University of Texas, Houston) [52]. All animals were raised under 12 hr light-dark cycles at 22-24°C with free access to food and water. All of the animal studies were approved by the Animal Ethics Committee of Tianjin Medical University. For the ectopic tumor model, adiponectin KO and WT mice were challenged with H7 (1×10^6^ cells per mouse) or Panc02 cells (2×10^6^ cells per mouse) in the flank. All of the tumor cell lines formed solid tumors in mice. Tumors were measured using digital vernier calipers, and their sizes were calculated as: TV= (Length) × (Width)^2^ /2 every three days. All of the mice were sacrificed four weeks after the initial implantation. Tumors, spleen, lymph gland, lung and liver tissues were collected and subjected to further analysis. For the orthotopic tumor model, an approximately 1-cm-long slit was cut into the side of the mice abdomen next to the pancreas, 5×10^5^ H7 cells were then injected into the pancreas, and then the slit was sutured. The mice were sacrificed two weeks after surgery, and the ascitic fluid and tumor metastasis was recorded. Tumors were collected, measured, weighed and imaged.

### Cell culture and reagents

Mouse ductal pancreatic adenocarcinoma cell line Panc02 was originally established by Corbett et al [22]. The highly metastatic Panc02-H7 cell line established using an in vivo selection method by Wang et al [23]. B16, Panc-1 and HepG2 cells were purchased from ATCC. Panc02, H7 and Panc-1 were cultured in DMEM (Gibic) supplemented with 10% fetal bovine serum (FBS) (Hyclone). HepG2 and B16 cells were cultured in 1640 (Gibic) supplemented with 10% fetal bovine serum (FBS) (Hyclone). All cells were cultured at 37°C in an atmosphere of 5% CO2 in air. The mouse and human full-length adiponectin produced from HEK293 cells were purchased from Bio-Way Biotechnology (Guang Zhou, China). Mouse adiponectin (ADP) ELISA Kit was purchased from Bio-Way Biotechnology (Guang Zhou, China), and performed following the manufacture’s instructions.

### Apoptosis and TUNEL assay

The Apoptosis Kit-Annexin V Alexa Fluor^®^ 488 & Propidium Iodide (Life Technologies) and TUNEL assay (TREVIGEN^®^) was performed following the manufacture’s instructions.

### Antibodies, western blotting analyses and immunoprecipitation

Antibodies for cleaved-caspase 3 (Asp175), caspase 3, phospho-AMPK (Thr172), AMPK, cyclin D1, acetylated-lysine, phospho-Akt (Ser473), phospho-GSK-3β (Ser9), phospho-cdc2 (Tyr15), cdc2, c-myc, phospho-Erk1/2 (Thr202/Tyr204) were purchased from Cell Signaling. β-catenin, Sirt-1, Sirt-2 were purchased from Abcam. PGC1α was purchased from Millipore. β-actin was purchased from Sungene. Whole cell lysate was prepared using RIPA lysis buffer in the presence of protease inhibitors. Protein concentrations were determined using the bicinchoninic acid (BCA) method (Biomed, Beijing, China). Total cell lysates were separated using 8%-12% SDS-PAGE, transferred onto PVDF membranes, and then detected using various primary antibodies. The antibody-antigen complexes were detected using the Chemiluminescent HRP Substrate (Millipore, MA, USA).

For immunoprecipitations, cells were lysed in lysis buffer (50 mM Tris-HCl at pH 7.4, 0.2 mM EDTA, 150 mM NaCl, 3% NP-40, 1 mM phenyl methylsulfonyl fluoride (PMSF) and, Protease inhibitor cocktail). Equal amounts of cell extracts were incubated with anti-PGC1α antibodies. Subsequent immunoblots were performed as described above.

### Cell death assay

H7 Cells were plated 1×10^5^ per well of a 12-well plate in duplicate plates for 12 h. Next, the cells were treated with mouse full-length adiponectin (0 or 10 μg/ml) along with doxorubicin (3 or 4 μM). After 24 h, all cells in the supernatant as well as adherent cells were collected from each well and resuspended in an equal volume of 1× PBS. The cells were diluted 1:10 in PBS and an equal volume of trypan blue dye was added to each sample. Next, the total number of cells and the total number of blue cells (dead cells that did not exclude the dye) were quantified using a hemocytometer. Cell death was presented as the percentage of dead cells compared with the total number of cells for each well.

### Cell proliferation

In this study, 20,000 H7 cells were plated in 12-well plates and treated with adiponectin (1-10 μg/ml) accompanied with doxorubicin (2 μM) in 2% FBS medium for 48 hours, Next, the cells were quantified and the numbers were plotted from three independent experimental wells.

### RNA isolation and RT-PCR

Total RNA was isolated using Trizol reagent (Invitrogen, Carlsbad, USA). Total RNA (2 μg) was used for synthesis of first-strand cDNA using M-MLV reverse transcriptase (Invitrogen, Beijing, China). Quantitative real-time PCR was performed using the SYBR green mix (Newbio industry, Beijing, China). The reactions were performed with a 7500 fast. Data were displayed as 2-ΔΔCt values and were representative of at least three independent experiments. Sequences of the RT-PCR primers were as follows (5′ - 3′):

GAPDH(CCATGTTCGTCATGGGTGTGAACCA and GCCAGTAGAGGCAGGGATGATGTTC);

AdipoR1(AATGGGGCTCCTTCTGGTAAC and GGATGACTCTCCAACGTCCCT);

AdipoR2(GCCAAACACCGATTGGGGT and GGCTCCAAATCTCCTTGGTAGTT);

IDH3A(ACAGGTGACAAGAGGTTTTGC and CTCCCACTGAATAGGTGCTTTG);

Ndufs3(TGGCAGCACGTAAGAAGGG and CTTGGGTAAGATTTCAGCCACAT); Cytc(GGTAACTGCAACTACTACTGTGG and CTTGATCTCGATCTTCGACATGG);

Cox5A(GCCGCTGTCTGTTCCATTC and GCATCAATGTCTGGCTTGTTGAA);

ATP5g1(TTCTCCAGCTCTGATTCGCTC and CCGGGAAATGACACTGGTCT);

ERRα(AGGTGGACCCTTTGCCTTTC and GGCATGGCGTACAGCTTCT).

### Constriction of recombinant lentivirus vectors and viral packaging

Replication-incompetent recombinant lentivirus expressing shRNAs were constructed using the PLKO.1 puro cloning vector (Addgene) according the Addgene’s instructions. PLKO.1 is a replication-incompetent lentiviral vector selected by the puromycin for the expression of shRNAs. The expression of shRNA is controlled by the human U6 promoter. A 21-mer oligonucleotide sequence was used against mouse AdipoR1, AdipoR2 and PGC1α. The oligonucleotides were annealed and ligated into the AgeI-EcoR1 sites of the shRNA vector PLKO.1-puro. To produce lentiviral particles, PLKO.1 shRNA plasmid or scramble shRNA plasmid (as control) with psPAX2 packing plasmid and pMD2.G envelope plasmid were transfected into 293T cells using X-tremeGENE HP DNA transfection Reagent (Roche). The biological titer of the purified virus was determined in 293T cells using serial dilution method. To select stable cell lines, viral supernatant was added to 10-cm dish for 24 hr, and then replaced with fresh medium for an additional 24 hr. Next, puromycin (8 μg/ml) was used to select stable cell lines. Sequences of the shRNAs were available as follows:

M(mouse)-shAdipoR1:5′-CCGGGAGACTGGCAACATCTGGACACTCGAGTGTCCAGATGTTGCCAGTCTCTTTTTG-3′;

M-shAdipoR2:5′-CCGGGCTTAGAGACACCTGTTTGTTCTCGAGAACAAACAGGTGTCTCTAAGCTTTTTG-3′;

M-shAMPK:5′-CCGGGAATCCTCATAGACCTTATTACTCGAGTAATAAGGTCTATGAGGATTCTTTTTG-3’;

M-shSirt1#1:5’-CCGGCTAGACCAAAGAATGGTATTTCTCGAGAAATACCATTCTTTGGTCTAGTTTTTG-3’;

M-shSirt1#2:5’-CCGGAGTGAGACCAGTAGCACTAATCTCGAGATTAGTGCTACTGGTCTCACTTTTTG-3’;

H(human)-shRNA-Sirt1#1:5’-CCGGGCAAAGCCTTTCTGAATCTATCTCGAGATAGATTCAGAAAGGCTTTGCTTTTTG-3’;

H-shRNA-Sirt1#2:5’-CCGGGATGATCAAGAGGCAATTAATCTCGAGATTAATTGCCTCTTGATCATCTTTTTG-3’;

M-shPGC1α#1:5′-CCGGCCAGAACAAGAACAACGGTTTCTCGAGAAACCGTTGTTCTTGTTCTGGTTTTTG-3′;

M-shPGC1α#2:5′-CCGGCGTGTGATTTACGTTGGTAAACTCGAGTTTACCAACGTAAATCACACGTTTTTG-3′.

### Caspase 3 activity assay

Cells were transfected using lenti-virus stably expressing caspase-3-like protease activation indicators (virus was a kind gift from Binghui Li). The cells were then treated with adiponectin or shRNA, and the fluorescence was recorded after 24 hours or other time points.

### Immunohistochemistry and Immunofluorescence

For immunohistochemical studies, 5μm paraffin-embedded tissue sections were deparaffinized and hydrated, followed by several washes with distilled water. The slides were then treated with antigen thermal retrieval in 0.01M citric acid- sodium citrate buffer (pH 6.0). After several washes with PBS, the slides were incubated with 2% bovine serum albumin diluted in PBS for 30 min at room temperature. Primary antibodies were dropped onto slides incubated at 4°C overnight. After washing with PBS, secondary antibodies (goat anti-rabbit IgG, Invitrogen) labeled with biotin were added and incubated at room temperature for 30 min. The slides were mounted with DAB (Cell Signaling) after washed with PBS.

For immunohistochemical studies, briefly, cells were fixed with 4% formaldehyde for 5min, and then incubated with 2% bovine serum albumin diluted in PBS for 30 min at room temperature. Cells were incubated with primary antibodies at 4°C overnight. After washing with PBS, secondary antibodies (donkey anti-rabbit IgG, Invitrogen) labeled with Alexa Fluor 555 were added and incubated at room temperature for 30 min. The slides were mounted with Fluomount-G containing DAPI (eBioscience) after PBS washing.

### Statistical analysis

All results were derived from at least three independent experiments. The data were expressed as the mean ± SEM and analyzed using a standard two-tailed Student’s t-test using EXCEL (Microsoft 2007). Differences with p < 0.05 were statistically significant.

## SUPPLEMENTARY MATERIAL AND FIGURES


