# Supplementary material for: Adiponectin promotes pancreatic cancer progression by inhibiting apoptosis via the activation of AMPK/Sirt1/PGC-1α signaling
Source: Oncotarget. 2014 May 13;5(13):4732–45. doi: 10.18632/oncotarget.1963 (PMC4148095; doi:10.18632/oncotarget.1963)
Supplement: Supplementary file 1 [file oncotarget-05-4732-s001.pdf]

Adiponectin promotes pancreatic cancer progression by inhibiting apoptosis via the activation of *AMPK*/Sirt1/PGC-1α signaling

Supplementary Material

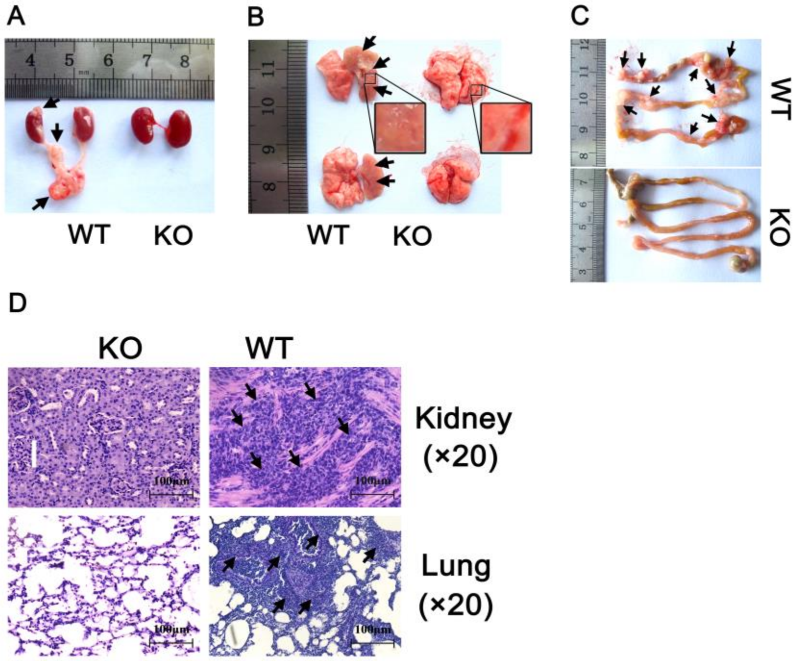

Supplementary Figure S1: Adiponectin deficiency can alleviate orthotopic pancreatic carcinoma metastasis. WT and APN KO mice were challenged with 5×10^5 H7 cells per mice (WT: n=8, KO: n=6). The mice were sacrificed two weeks after inoculation. The ascitic fluid and tumor metastasis was recorded. (A, B, and C) The kidney (A), pulmonary (B), and intestine (C) metastasis were imaged. (D) Representative H&E stain of kidney and lung metastasis. Scale bars, 100 μm.

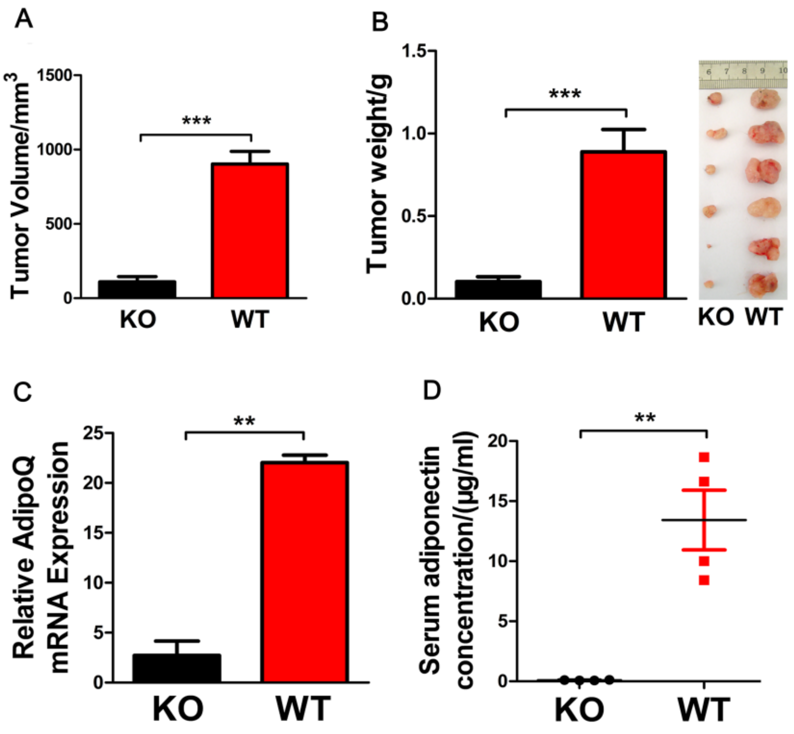

Supplementary Figure S2: Adiponectin deficiency inhibits pancreatic cancer cell growth *in vivo*. (A, B) WT and APN KO mice were subcutaneously challenged with Panc02 cells (2×10^6 cells per mice, n=8 per group) in the flank. Four weeks after inoculation, the mice were sacrificed, and the Panc02 tumors were collected, measured, and weighed and imaged. (C) Relative APN mRNA level in adipose tissue from APN KO and WT mice. (D) The serum APN level was measured using an ELISA kit. The values represent the mean ± SEM of three independent experiments performed in triplicate. **: $0.001<p<0.01$; ***: $p<0.001$.

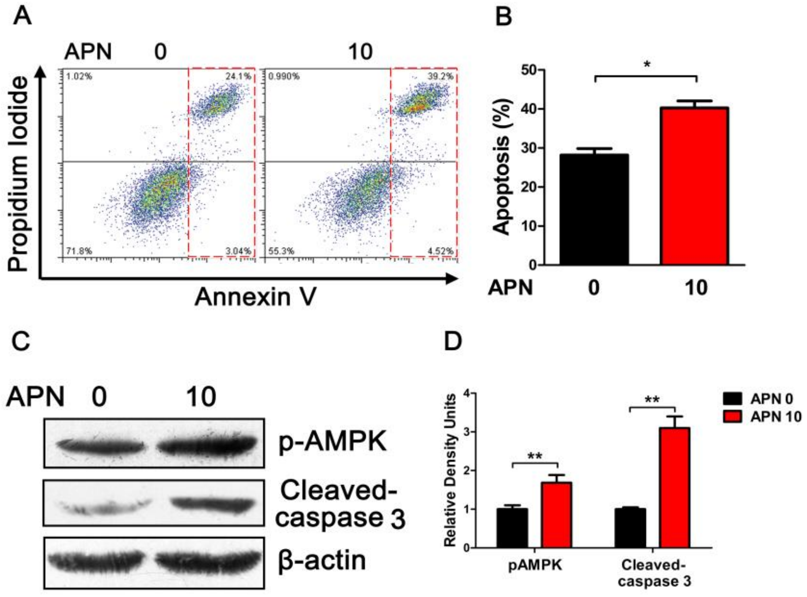

Supplementary Figure S3: Adiponectin is pro-apoptotic for HepG2 cells. (A) HepG2 cells were treated with adiponectin (0 or 10 μg/ml) in 2% FBS medium for 24 hours, and apoptosis was then detected using Annexin V. (B) Quantification of apoptosis described in A. (C) Cells were treated as described in A, and the levels of p-AMPK and cleaved-caspase 3 were analyzed using immunoblotting. (D) Quantitative analysis of the data shown in C. The values represent the mean ± SEM of three independent experiments performed in triplicate. **: 0.001<p<0.01; ***: p<0.001.

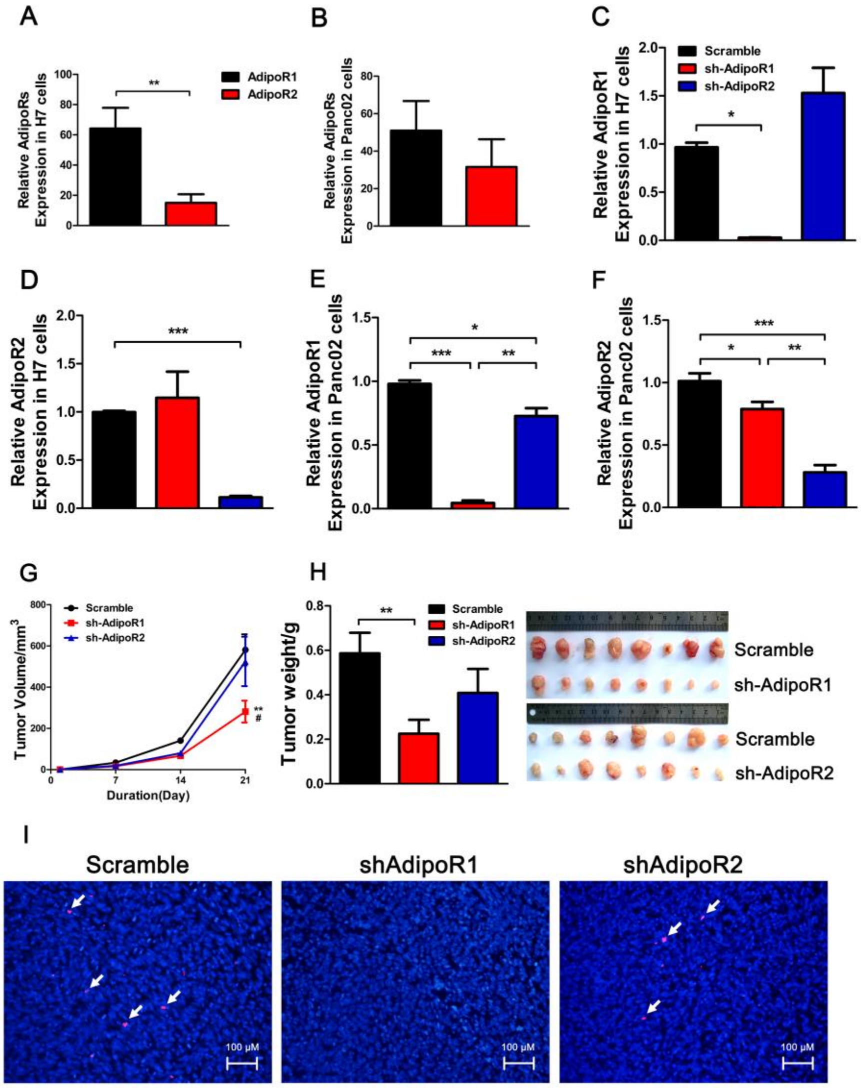

Supplementary Figure S4: Suppression of AdipoR1 promotes apoptosis and inhibits proliferation. (A, B) RT-PCR analysis of AdipoR1 and AdipoR2 mRNA in H7 and Panc02 cells. (C-F) RT-PCR analysis of AdipoR1 and AdipoR2 mRNA in AdipR1 knockdown H7 (C) or Panc02 cells (E) and AdipoR2 knockdown H7 (D) or Panc02 cells (F). (G, H) Tumor formation assay results after knockdown of AdipoRs. 2×10^6 Panc02 cells with the indicated expression conditions of AdipoRs were injected subcutaneously into 6- to 8- week-old C57BL/6 mice. The tumor size was monitored once a week for three weeks. Next, the mice were sacrificed, and the tumors were resected and weighed. Representative tumor volume of Panc02 cells. Tumor weights and images of the excised tumors (n=8 mice per group, **: shAdipoR1 and scramble; $^{\#}$: shAdipoR1 and shAdipoR2). (I) Immunofluorescence of Ki-67 in Panc02 tumor sections after AdipoR knockdown. Scale bars, 100 μm. The values represent the mean ± SEM of three independent experiments performed in triplicate. * or $^{\#}$: $0.01<p<0.05$; **: $0.001<p<0.01$; ***: $p<0.001$.

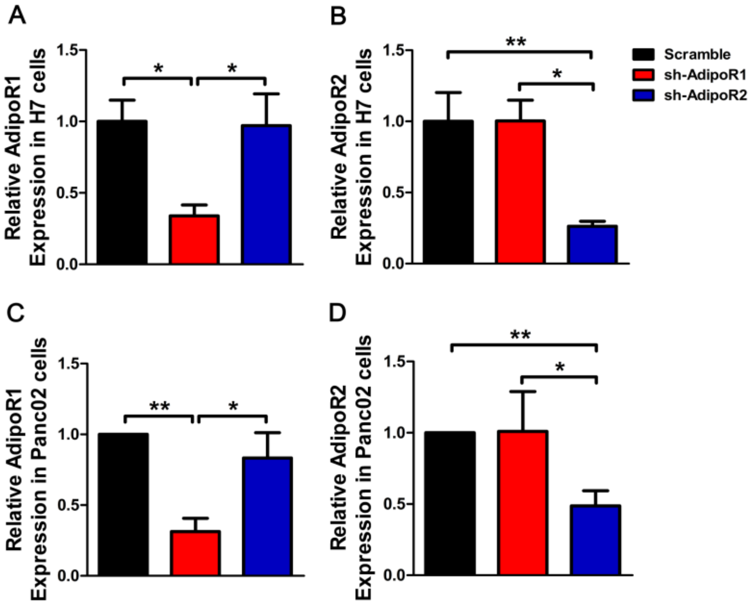

Supplementary Figure S5: Expression level of AdipoR1 and AdipoR2 in H7 and Panc02 cells. (A-D) AdipoR1 and AdipoR2 knockdown H7 and Panc02 cells (1×10^6 and 2×10^6 cells) were injected subcutaneously into 6- to 8- week-old C57BL/6 mice, and the mice were sacrificed after 3 weeks. Expression level of AdipoR1 and AdipoR2 in H7 (A, B) and Panc02 cells (C, D) were detected using RT-PCR.

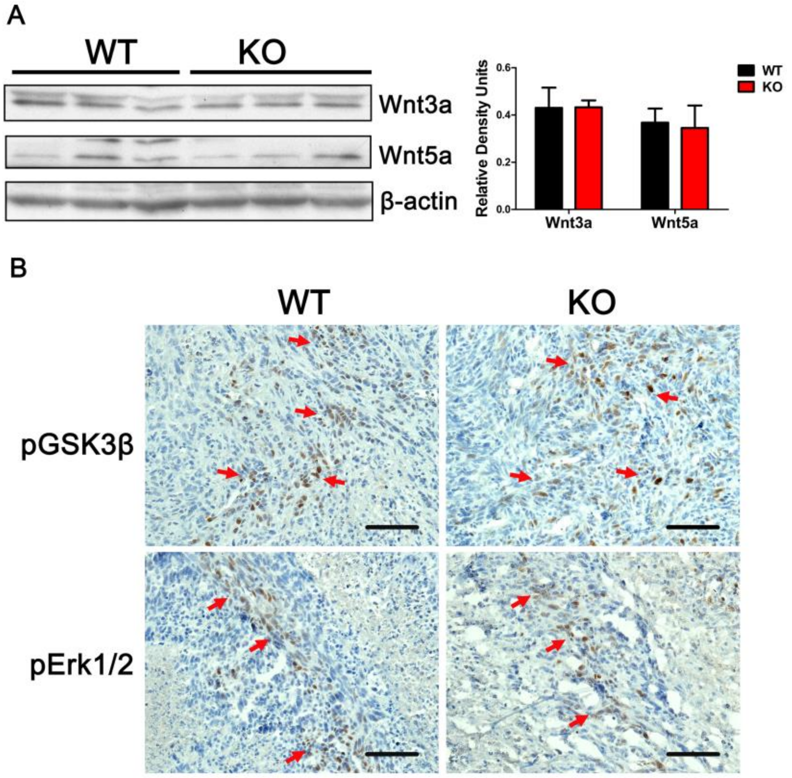

Supplementary Figure S6: Adiponectin does not influence Wnt3a and Wnt5a signaling. (A) H7 tumor lysates from APN KO and WT mice were analyzed using immunoblotting with the indicated antibodies. (B) H7 tumor section from APN KO and WT mice were stained with pGSK3β and pErk1/2 antibodies. Scale bars, 100 μm. The values represent the mean ± SEM of three independent experiments performed in triplicate.
